# Dietary patterns and their relationship with hepatobiliary health in patients with nonalcoholic fatty liver disease and gallstone disease

**DOI:** 10.3389/fnut.2026.1789238

**Published:** 2026-08-11

**Authors:** Ziqing Yu, Wen He

**Affiliations:** Department of Geriatrics, The First Affiliated Hospital of Sun Yat-Sen University, Guangzhou, Guangdong, China

**Keywords:** dietary patterns, gallstone disease, hepatobiliary health, nonalcoholic fatty liver disease, oxidative stress, western diet

## Abstract

**Background:**

Dietary habits are important factors of metabolic and hepatobiliary health. However, there is still a lack of research on the relationships between gallstone disease, nonalcoholic fatty liver disease (NAFLD), and regular eating habits.

**Objective:**

To investigate the associations between dietary patterns and hepatobiliary outcomes, including NAFLD, gallstone disease, and related biochemical markers, in adults who visit tertiary-care clinics.

**Methods:**

This retrospective observational association study involved 600 adults aged 20–70 years with a principal component analysis of a validated food frequency questionnaire and categorized them into healthy (*n* = 285) and Western (*n* = 315) diets. Hepatobiliary health was assessed by anthropometric measurements, fasting biochemical (alanine aminotransferase, fasting glucose, lipid profile, malondialdehyde) measurements, and abdominal ultrasonography. The odds ratios (ORs) of NAFLD and gallstone disease were estimated using logistic regression to control age, sex and BMI. The effect modification of overall and central adiposity was studied through stratified analyses.

**Results:**

Higher BMI, a wider waist circumference, more energy intake, and less physical activity were all related to following a Western diet (all *p* < 0.001). In crude and partially adjusted models, Western dietary patterns were associated to increased odds of NAFLD and gallstone disease; however, these relationships were not statistically significant after additional adjustment for waist circumference, total energy intake, and physical inactivity (NAFLD: OR = 1.37, 95% CI: 0.68–2.75, *p* = 0.384; gallstone disease: OR = 1.83, 95% CI: 0.93–3.61, *p* = 0.080). Participants following a Western dietary pattern exhibited higher levels of ALT, triglycerides, total cholesterol, fasting glucose, and malondialdehyde, along with lower HDL-C concentrations (all *p* < 0.001). Most of the participants with overweight, obesity, or abdominal obesity showed the strongest associations.

**Conclusion:**

These results shed more light on the association between dietary patterns and hepatobiliary health. More research on dietary-pattern-oriented approaches is necessary to fully comprehend their potential impact in hepatobiliary health.

## 1 Introduction

The hepatobiliary system, which consists of the biliary tract, gallbladder, and liver, is essential for preserving metabolic homeostasis. Non-alcoholic fatty liver disease (NAFLD), cirrhosis, hepatocellular carcinoma (HCC), gallstone disease, and recently renamed metabolic dysfunction-associated steatotic liver disease (MASLD) are disorders that impact this system and significantly contribute to the global disease burden. Dietary shifts toward energy-dense, nutrient-poor diets and more sedentary lifestyles have been linked to the rising incidence of these disorders with nutrition thought to be a significant modifiable factor in hepatobiliary health (1). Dietary patterns have become the focus of more recent nutritional studies since dietary patterns more accurately reflect regular eating behavior and cumulative nutritional intake than individual components. The epidemiological and clinical studies are pointing toward the evidence that the effect of following healthy dietary patterns, including Mediterranean, DASH and plant-based diets, is correlated with better liver functioning, less hepatic fat build-up, and favorable bile composition. On the other hand, hepatic steatosis, inflammation, and biliary dysfunction have been repeatedly linked to Western-style dietary patterns that are high in refined carbs, saturated fats, and ultra-processed foods (2).

Western and pro-inflammatory eating habits have been repeatedly linked to higher risks of NAFLD, hepatic steatosis, hepatocellular carcinoma, and other hepatobiliary diseases. On the other hand, more favorable liver-related outcomes, better metabolic profiles, and reduced hepatic fat deposition have all been linked to Mediterranean, prudent and plant-rich dietary patterns. All of these outcomes point to the essential role that total dietary quality plays in hepatobiliary health and disease progression (3–5). Gallstone disease is also strongly associated with dietary habits. Diets heavy in refined carbohydrates and animal fats have been linked to an a greater likelihood of gallstones, while diets high in fiber, fruits, vegetables, and unsaturated fats have been linked to a reduced risk (6–8). Furthermore, gallstone disease and NAFLD often coexist and may have similar nutritional and metabolic mechanisms (9).

The association between food habits and hepatobiliary health could be explained by a number of biological processes. Diets heavy in refined carbohydrates and saturated fats may increase oxidative stress, insulin resistance, hepatic *de novo* lipogenesis, and chronic low-grade inflammation—all of which are important contributors to liver fibrosis and steatosis. On the other hand, high-fiber, antioxidant, and unsaturated fatty acid diets raise insulin sensitivity, modulate bile acid metabolism and lowers lipid accumulation in the liver (10). Inflammation is one of the primary paths as a study found that the highest scores of Dietary Inflammatory Index were the predictors of the risk of gallstones (11). Also, bile acids are metabolic signaling molecules, which are highly impacted by the composition of the diet via nuclear receptors, namely FXR and TGR5. Changes in these signaling pathways have an influence on lipid metabolism, glucose homeostasis, and the motility of the gallbladder. The diet-induced alterations in gut microbiota also regulate the metabolism of the bile acids and systemic inflammation, which further supports the role of the gut-liver-gallbladder axis in the hepatobiliary health (10).

Despite substantial evidence linking dietary habits to hepatobiliary disorders, there are still a number of significant gaps in the body of current research. The majority of prior research has mostly concentrated on specific hepatobiliary outcomes instead of assessing NAFLD, gallstone disease, and associated metabolic abnormalities collectively within the same cohort. Since both illnesses often share shared metabolic and nutritional risk factors, a more comprehensive evaluation may offer a better understanding of the association between eating habits and overall hepatobiliary health. Thus, the purpose of this study was to examine the relationships between dietary habits and hepatobiliary outcomes, such as gallstone disease, nonalcoholic fatty liver disease (NAFLD), and associated biochemical markers in people who visit tertiary care clinics.

## 2 Materials and methods

### 2.1 Study design

This research was carried out as a retrospective observational analysis utilizing regularly obtained clinical and dietary information from adults assessed at tertiary-care outpatient clinics between January 2020 and December 2023. Data were retrieved from electronic health records and the institutional dietary databases. This research was approved by the Ethics Committee of The First Affiliated Hospital of Sun Yat-Sen University, approval number: Ethical [2025] 483.

### 2.2 Study population

**Figure 1:**
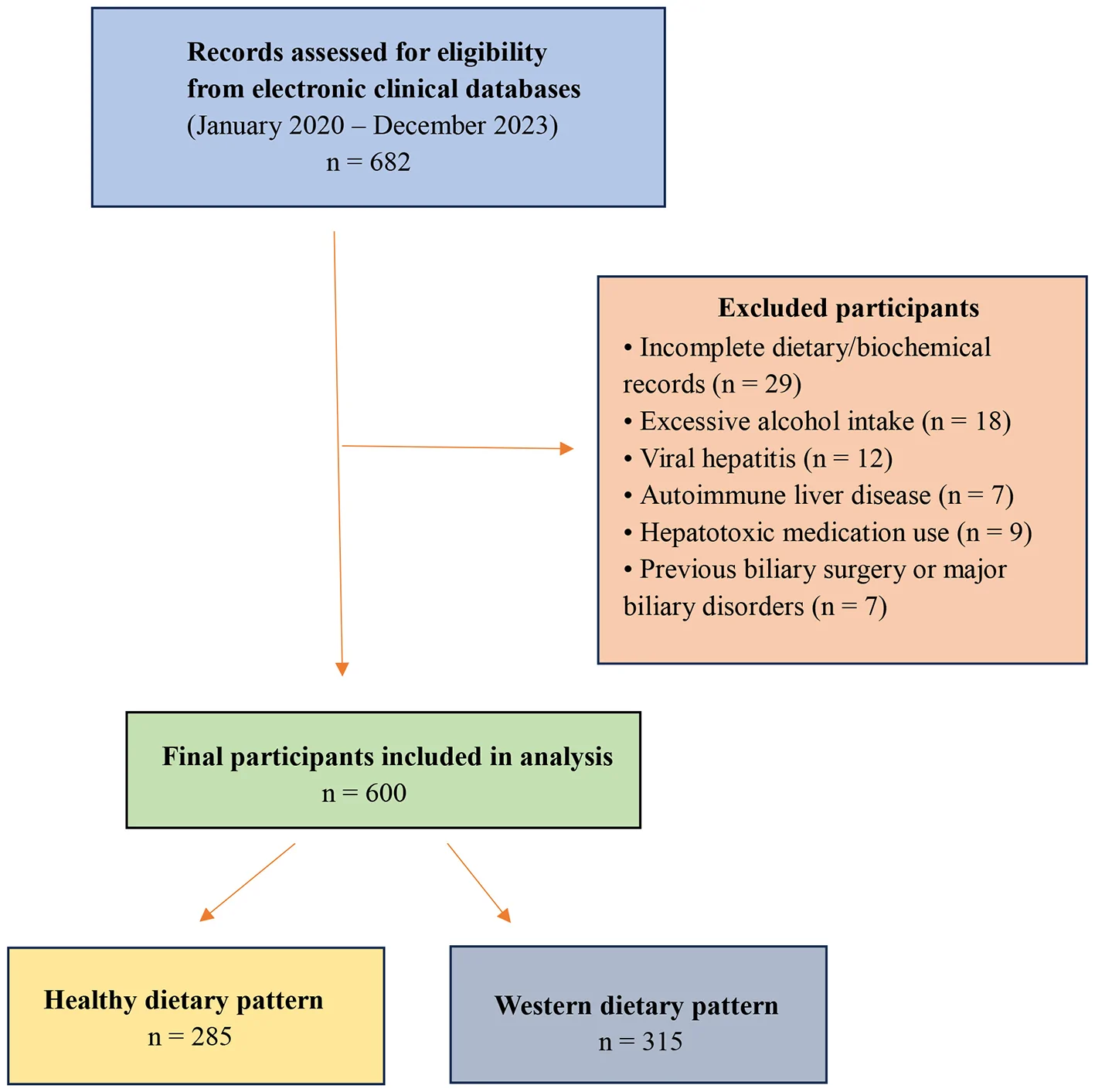
Flow diagram of participant selection.

Health records of 682 subjects were initially screened. Individuals were considered if they were between the ages of 20 and 70 years and had detailed data on dietary consumption, anthropometric measurements, fasting biochemical markers, and abdominal ultrasonography. Participants with reported excessive alcohol intake (>20 g/day for women and >30 g/day for men), viral hepatitis, autoimmune liver condition, or usage of hepatotoxic medicines were not included (Figure 1).

### 2.3 Dietary assessment and dietary pattern derivation

Habitual dietary intake was measured at the time of clinical examination employing a validated semi-quantitative food frequency questionnaire (FFQ) encompassing intake over the previous 6 months. Standard serving sizes and predetermined intake frequency options—from infrequently or never to many times daily—were included in the questionnaire. For the purpose of dietary pattern analysis, reported consumption frequencies were transformed into average daily intake values for each food item and then separated into 16 nutritionally comparable food groups. The FFQ demonstrated acceptable reproducibility and validity in the original validation study with intraclass correlation coefficients ranging from 0.49 to 0.71 and energy-adjusted correlation coefficients ranging from 0.36 to 0.75 for major nutrients and food groups, suggesting that it was appropriate for evaluating typical dietary intake in epidemiological studies (12).

Dietary patterns were determined via principal component analysis (PCA) based on 16 FFQ-derived food groups, such as fruits, vegetables, whole grains, legumes, seafood, nuts/seeds, unsaturated oils, low-fat dairy, refined cereals, red/processed meat, fried foods, sweets/desserts, sugar-sweetened beverages, fast food, salty snacks, and high-fat dairy. PCA was carried out utilizing the correlation matrix followed by orthogonal varimax rotation to enhance interpretability. Eigenvalues larger than 1.5, a visual examination of the scree plot, and the biological interpretability of the rotated factor-loading structure were the criteria used to retain components. Food groups were deemed significant contributors to each dietary pattern if their absolute rotational factor loadings were 0.40 or above. The two eating patterns that were kept accounted for 29.1% of the overall variation in food intake. Supplementary Figure S1 displays the scree plot, and Supplementary Table S1 provides the rotated factor-loading matrix. Each participant's standardized PCA component score was computed, and they were categorized based on the eating pattern with the highest dominant component score.

Two main patterns of eating were found. Increased consumption of fruits, vegetables, whole grains, legumes, seafood, and unsaturated fats was the hallmark of the healthy pattern. Higher consumption of refined cereals, red and processed meats, fried foods, sweets, and beverages with added sugar were characteristics of the Western pattern.

### 2.4 Anthropometric and lifestyle variables

Height, weight, and waist circumference were evaluated by trained clinical personnel utilizing established methods. Body mass index (BMI) was computed as weight (kg) divided by height squared (m^2^). A waist circumference of at least 94 cm for men and 80 cm for women was considered abdominal obesity. The International Physical Activity Questionnaire (IPAQ-SF), a standardized questionnaire that tracks walking, moderate-intensity activity, vigorous-intensity activity, and sitting time during the preceding seven days, was used to measure physical activity status. Participants were classified as physically active or physically inactive for analysis based on IPAQ scoring criteria.

### 2.5 Laboratory measurements

Following a 10–12 h overnight fast, fasting venous blood samples were collected and subjected to established quality-controlled methods at the hospital laboratory. Automated enzymatic tests were used to evaluate serum alanine aminotransferase, triglycerides, total cholesterol, and HDL cholesterol. The glucose oxidase method was used to gauge plasma glucose during fasting. The thiobarbituric acid reactive compounds assay was used to measure malondialdehyde (MDA), a sign of lipid peroxidation.

### 2.6 Assessment of NAFLD and gallstone disease

Abdominal ultrasonography was used to detect NAFLD and gallstone disease during regular clinical evaluation by skilled radiologists who were not involved in the dietary assessment. NAFLD was identified based on ultrasound characteristics of hepatic steatosis, such as increased liver echogenicity, vascular blurring, and deep attenuation of the ultrasound signal after eliminating individuals with excessive alcohol consumption, viral hepatitis, autoimmune liver disease, or other chronic liver disorders. Echogenic mobile entities with posterior acoustic shadowing inside the gallbladder were indicative of gallstone disease. To increase the accuracy of outcome classification, participants with prior cholecystectomy, biliary blockage, biliary cancer, primary sclerosing cholangitis, or other significant biliary tract illnesses were eliminated.

### 2.7 Statistical analysis

Categorical data are presented as frequencies and percentages, while continuous factors are stated as mean ± standard deviation. Independent *t*-tests or chi-square tests were used to evaluate dietary group differences. Independent t-tests or chi-square testing were employed to examine dietary group differences, including baseline laboratory values. Adjusted linear regression models that controlled for age, sex, BMI, waist circumference, total calorie consumption, and physical inactivity were used to further assess biochemical marker variations between dietary patterns. Odds ratios (ORs) and 95% confidence intervals (CIs) for gallstone disease and NAFLD were calculated using logistic regression models based on dietary patterns. BMI was not regarded as a confirmed mediator, but rather as a covariate associated with obesity. Progressive logistic regression models were developed to assess the relationship between dietary patterns and hepatobiliary outcomes. Model 5 was regarded as the main inferential model since it included all predetermined demographic, anthropometric, and lifestyle-related factors found in the dataset—such as age, sex, BMI, waist circumference, total energy intake, and physical inactivity. Physical inactivity, total calorie intake, and waist circumference were included since they are strongly associated with dietary habits and hepatobiliary health. Their exact causal involvement could not be ascertained due to the study's retrospective observational design. Stratified tests based on BMI and abdominal obesity were used to investigate effect modification by adiposity. A threshold of *p* < 0.05 was used for statistical significance. Furthermore, continuous PCA-derived eating pattern scores were used for sensitivity analyses. Logistic regression was utilized to assess relationships per 1-SD rise in Healthy and Western dietary pattern scores.

## 3 Results

### 3.1 Baseline characteristics

The investigation comprised 600 individuals in total, of whom 285 were categorized as adhering to a healthy dietary pattern and 315 as following a Western dietary pattern. Baseline features are reported in Table 1. Individuals in the Western group were marginally older than those in the Healthy group (*p* = 0.008), while the percentage of female did not differ substantially between groups (*p* = 0.409). Anthropometric measurements showed significant variations. The Western group's mean BMI was greater than the Healthy group's (*p* < 0.001). In a similar vein, subjects who followed a Western dietary pattern had larger waist circumferences than those who followed a healthy pattern. Additionally, the Western group's daily energy intake was substantially higher than that of the Healthy group. Western-pattern individuals were more likely to be physically inactive (58.1%) than Healthy-pattern adults (21.8%, *p* < 0.001). There were variations in the prevalence of diseases among dietary categories. In the Western group, 28.9% of subjects had NAFLD, as opposed to 12.3% in the Healthy group (*p* < 0.001). Gallstone disease was found in 14.4% of people with a healthy pattern and 26.3% of those with a Western pattern (*p* < 0.001).

**Table 1:** Baseline characteristics by dietary pattern.

Variable	Healthy pattern (*n* = 285)	Western pattern (*n* = 315)	*p*-value
Age (years)	44.96 ± 8.41	46.69 ± 7.59	0.008
Female *n* (%)	127 (44.6)	151 (47.9)	0.409
BMI (kg/m^2^)	23.87 ± 2.47	27.57 ± 3.36	< 0.001
Waist circumference (cm)	84.92 ± 8.53	92.01 ± 9.55	< 0.001
Total energy intake (kcal/day)	2201 ± 276	2795 ± 335	< 0.001
Physically inactive *n* (%)	62 (21.8)	183 (58.1)	< 0.001
Alanine aminotransferase (U/L)	30.17 ± 4.99	44.72 ± 7.18	< 0.001
Malondialdehyde (nmol/ml)	2.30 ± 0.39	3.24 ± 0.51	< 0.001
Fasting blood sugar (mg/dl)	96.52 ± 8.31	104.93 ± 9.41	< 0.001
Triglycerides (mg/dl)	129.40 ± 18.65	153.30 ± 21.09	< 0.001
Total cholesterol (mg/dl)	186.81 ± 17.96	202.97 ± 19.84	< 0.001
HDL cholesterol (mg/dl)	55.19 ± 6.21	42.29 ± 5.47	< 0.001
NAFLD *n* (%)	35 (12.3)	91 (28.9)	< 0.001
Gallstone disease *n* (%)	41 (14.4)	83 (26.3)	< 0.001

Values are presented as mean ± SD or n (%). Laboratory measurements were further examined by employing adjusted linear regression models that controlled for age, sex, waist circumference, BMI, total calorie intake, and physical inactivity, as reported in Table 4.

### 3.2 Dietary patterns and NAFLD

In the unadjusted model, individuals who followed a Western dietary pattern had significantly greater chances of NAFLD than those who followed a healthy dietary pattern (OR = 2.90, *p* < 0.001). Even after controlling for age and sex (OR = 2.77, *p* < 0.001) and BMI (OR = 2.03, *p* = 0.006), the association was still statistically significant. Nevertheless, the found association was weakened and no longer statistically significant after additional modifications for physical inactivity, total energy intake, and waist circumference (Table 2).

**Table 2:** Dietary patterns and NAFLD risk.

Model	Dietary pattern	OR	95% CI	*p*-value
Model 1	Healthy	Reference	—	—
	Western	2.90	1.89–4.46	< 0.001
Model 2	Healthy	Reference	—	—
	Western	2.77	1.79–4.26	< 0.001
Model 3	Healthy	Reference	—	—
	Western	2.03	1.23–3.35	0.006
Model 4	Healthy	Reference	—	—
	Western	1.32	0.75–2.33	0.341
Model 5	Healthy	Reference	—	—
	Western	1.37	0.68–2.75	0.384

Model 1: Unadjusted. Model 2: Adjusted for age and sex. Model 3: Model 2 + BMI. Model 4: Model 3 + waist circumference. Model 5: Model 4 + total energy intake and physical inactivity.

### 3.3 Dietary patterns and gallstone disease

Compared to healthy eating habits, Western eating patterns were associated with elevated risks of gallstone disease. Participants who followed a Western eating pattern were more than twice as likely to develop gallstone disease in the unadjusted model (OR = 2.13, *p* < 0.001). When age, sex, and BMI were taken into account, the association was still statistically significant (OR = 1.67, *p* = 0.040). The found correlations were weakened and not statistically significant after additional adjustment for waist circumference, energy consumption, and physical inactivity (Table 3).

**Table 3:** Dietary patterns and gallstone disease risk.

Model	Dietary pattern	OR	95% CI	*p*-value
Model 1	Healthy	Reference	—	—
	Western	2.13	1.41–3.22	< 0.001
Model 2	Healthy	Reference	—	—
	Western	2.07	1.37–3.15	< 0.001
Model 3	Healthy	Reference	—	—
	Western	1.67	1.03–2.73	0.040
Model 4	Healthy	Reference	—	—
	Western	1.50	0.87–2.58	0.147
Model 5	Healthy	Reference	—	—
	Western	1.83	0.93–3.61	0.080

Model 1: Unadjusted. Model 2: Adjusted for age and sex. Model 3: Model 2 + BMI. Model 4: Model 3 + waist circumference. Model 5: Model 4 + total energy intake and physical inactivity.

### 3.4 Biochemical markers by dietary pattern

Table 4 displays variations in biochemical markers among food categories. Individuals with Western patterns had higher levels of alanine aminotransferase (ALT) than those with healthy patterns (*p* < 0.001). Furthermore, the Western dietary group showed increased levels of biomarkers associated with oxidative stress. The Western dietary group had 40% greater levels of malondialdehyde (MDA) (*p* < 0.001), which suggests increased oxidative damage and lipid peroxidation. Those eating a Western diet showed signs of metabolic dysregulation. The Western group had increased fasting blood sugar readings than the Healthy group (*p* < 0.001). Individuals with a Western pattern had higher triglyceride levels than those with a healthy pattern (*p* < 0.001). Additionally, the Western group had greater total cholesterol (*p* < 0.001). Conversely, high-density lipoprotein cholesterol (HDL-C), a protective lipid fraction, was substantially decreased in the Western dietary category (*p* < 0.001).

**Table 4:** Biomarker associations with dietary patterns.

Biomarker	Healthy, Mean ±SD	Western, Mean ±SD	Unadjusted mean difference	Adjusted β	95% CI	*p*-value
Alanine aminotransferase (U/L)	30.17 ± 4.99	44.72 ± 7.18	+14.55	+14.27	12.60–15.95	< 0.001
Malondialdehyde (nmol/ml)	2.30 ± 0.39	3.24 ± 0.51	+0.93	+0.92	0.80–1.04	< 0.001
Fasting blood sugar (mg/dl)	96.52 ± 8.31	104.93 ± 9.41	+8.41	+6.93	4.55–9.31	< 0.001
Triglycerides (mg/dl)	129.40 ± 18.65	153.30 ± 21.09	+23.90	+24.51	19.14–29.87	< 0.001
Total cholesterol (mg/dl)	186.81 ± 17.96	202.97 ± 19.84	+16.16	+15.63	10.55–20.71	< 0.001
HDL cholesterol (mg/dl)	55.19 ± 6.21	42.29 ± 5.47	−12.89	−12.64	−14.20 to −11.08	< 0.001

Adjusted for age, sex, BMI, waist circumference, energy intake, and physical inactivity. Adjusted β represents the unstandardized regression coefficient (mean difference).

### 3.5 Stratified analyses by adiposity

After controlling for age and sex, the Western eating pattern was associated with NAFLD in individuals with BMI ≥25 kg/m^2^ (adjusted OR: 2.82, 95% CI: 1.35–5.90, *p* = 0.006). In this category, however, there was no discernible correlation between gallstone disease and the Western eating pattern (adjusted OR: 1.38, 95% CI: 0.57–3.37, *p* = 0.480). Neither NAFLD (p = 0.453) nor gallstone disease (*p* = 0.811) showed statistically significant interaction terms between dietary pattern and BMI status. Moreover, after controlling for age and sex, the Western eating pattern was associated with gallstone disease (adjusted OR: 3.21, 95% CI: 1.54–6.66, *p* = 0.002) and NAFLD (adjusted OR: 9.44, 95% CI: 3.05–29.25, *p* < 0.001) among individuals with abdominal obesity. For NAFLD, the interaction between eating habits and abdominal obesity was statistically significant (p = 0.002), but not for gallstone disease (*p* = 0.059) (Table 5).

**Table 5:** Western pattern and disease risk in high-risk adiposity groups.

Adiposity group	Outcome	Healthy, *n*	Western, *n*	Cases healthy/Western	Western adjusted OR	95% CI7	*p*-value	Interaction p-value
BMI ≥ 25 kg/m^2^ (overweight/obese)	NAFLD	95	249	16/75	2.82	1.35–5.90	0.006	0.453
BMI ≥ 25 kg/m^2^ (overweight/obese)	Gallstone disease	95	249	24/75	1.38	0.57–3.37	0.480	0.811
Abdominal obesity (men ≥ 94 cm; women ≥ 80 cm)	NAFLD	126	233	31/74	9.44	3.05–29.24	< 0.001	0.002
Abdominal obesity (men ≥ 94 cm; women ≥ 80 cm)	Gallstone disease	126	233	26/62	3.21	1.54–6.66	0.002	0.059

### 3.6 Sensitivity analysis

Sensitivity analysis was carried out utilizing PCA-derived dietary pattern scores as continuous variables. Increased Western dietary pattern scores revealed a non-significant tendency toward increased odds of NAFLD, while elevated Healthy dietary pattern scores indicated a non-significant link with decreased odds of NAFLD after controlling for age, sex, and BMI. Higher Western eating pattern scores were significantly related with increased probabilities of gallstone disease, while greater Healthy dietary pattern scores were significantly associated with lower odds (Table 6).

**Table 6:** Sensitivity analysis using continuous PCA-derived dietary pattern scores.

Outcome	Dietary pattern score	Adjusted OR	95% CI	*p*-value
NAFLD	Healthy score	0.880	0.707–1.095	0.250
NAFLD	Western score	1.197	0.968–1.480	0.096
Gallstone disease	Healthy score	0.781	0.628–0.971	0.026
Gallstone disease	Western score	1.238	1.004–1.528	0.046

## 4 Discussion

In this research, particular dietary habits were firmly correlated with variation in anthropometric traits, metabolic condition, prevalence of liver disease, prevalence of gallstones, and biochemical indicators of hepatobiliary diseases. All of these results point to the possible significance of habitual eating habits for hepatobiliary health and indicate that Western-style eating habits may be connected to both biliary and hepatic dysfunction via similar inflammatory and metabolic processes. People following a Western dietary pattern were older, were characterized by a high body mass index, larger waist circumference, and increased total energy intake than healthy dietary pattern. These results are in line with earlier research showing that in communities with metabolic disorders, Western eating patterns are often linked to increased caloric consumption, central obesity, and decreased levels of physical activity. The clustering of unfavorable lifestyle behaviors frequently described in nutritional epidemiology studies is further supported by the significantly greater incidence of physical inactivity among persons who follow a Western eating pattern (13).

The association between dietary quality and hepatobiliary health is demonstrated by the increased incidence of NAFLD and gallstone disease among individuals who adhere to a Western diet. These findings are mostly in line with population-based research showing assocations between poor eating practices, obesity-associated phenotypes, hepatic steatosis, and gallstone disease (14). According to the current study, those who followed a Western eating pattern had nearly three times the odds of developing non-alcoholic fatty liver disease (NAFLD) in the unadjusted model. This association was still statistically significant when age, sex, and BMI were taken into account. However, following further adjustment for waist size, total energy intake, and physical inactivity, the found relationships were weakened and no longer statistically significant. These results imply that factors related to lifestyle and obesity may account for a considerable portion of the observed association. Therefore, the findings do not show an independent relationship between Western dietary patterns and NAFLD following thorough adjustment. Hepatic lipid buildup, insulin resistance, oxidative stress, and activation of the inflammatory system are among the possible pathways suggested in earlier experimental and clinical literature (15). These biological processes, however, should be regarded as reasonable theories rather than established mechanisms since they were not directly assessed in the current investigation.

Gallstone disease showed a similar pattern. In crude and partially adjusted analyses, a Western dietary pattern was related to increased risks of gallstone disease; however, following additional adjustment for waist circumference, total energy intake, and physical inactivity, the association was weakened and no longer statistically significant. These results suggest that lifestyle and obesity-related traits that frequently cluster with Western eating habits may have an impact on the observed correlation. As a result, the current findings do not demonstrate an independent relationship between Western dietary patterns and gallstone disease in the fully adjusted analysis. These results are aligned with the established evidence of the linkage between high levels of refined carbohydrates, animal fats, and low fiber intake with the formation of gallstones due to changes in the composition of bile and the loss of gallbladder motility (16). These observations are further supported by large-scale cohort studies that were conducted recently. A study revealed that poor eating habits are major contributors to the development of cholelithiasis, and the dietary factors interact with genetic predisposition (17). Likewise, meta-analyses of nutritional risk factors have always found unhealthy dieting to be a nutritional risk factor that is modifiable and predisposes biliary disease (18). When taken as a whole, these results are in line with earlier research that suggests Western dietary practices may influence the formation of gallstones by changing the composition of bile and regulating metabolism. Nevertheless, this study did not evaluate bile acid metabolism or associated molecular processes directly.

The sensitivity analysis utilizing continuous PCA-derived dietary pattern scores additionally reinforced a careful interpretation. When dietary patterns were evaluated via continuous PCA-derived scores, no significant relationships were found between either dietary pattern score and NAFLD after adjustment. This implies that the association between dietary pattern and non-alcoholic fatty liver disease (NAFLD) was not as strong as the categorical analysis suggested and should thus be read cautiously. Conversely, the relationships found for gallstone disease continued to be statistically significant in the continuous-score analysis even though the effect sizes were but modest and borderline.

The Western dietary pattern was also associated with an unfavorable biochemical profile such as increased alanine aminotransferase, increased malondialdehyde, dyslipidemia, poor glycemic control. Hepatocellular injury is indicated by elevated ALT levels and is typical in metabolic liver disease, whereas increased oxidative stress and lipid peroxidation are indicated by the presence of increased malondialdehyde levels. The noticed biochemical variations could be a reflection of more general metabolic disorders associated with Western food habits. Hepatobiliary dysfunction may be influenced by oxidative stress, inflammatory reactions, and altered bile acid metabolism, according to earlier research. However, this investigation did not explicitly evaluate these pathways, and the current results should be evaluated in light of an observational study methodology (19).

Limited evidence of adiposity-related effect modification was seen in the stratified analysis. For both NAFLD and gallstone disease, there was no discernible relationship between dietary pattern and BMI status. On the other hand, for NAFLD, a significant interaction between dietary pattern and abdominal obesity was found; however, the comparable interaction for gallstone disease was not statistically significant. These results imply that central adiposity may have an impact on the observed associations, especially for NAFLD. This finding aligns with other research demonstrating that visceral adiposity, and central obesity are more significantly linked to NAFLD than BMI-defined obesity alone (20, 21). Importantly, the results among patients with abdominal obesity may suggest a possible interaction between visceral adiposity and dietary variables in hepatobiliary dysfunction although the underlying biological processes were not particularly investigated in this study.

The combined incidence of NAFLD and gallstone disease in the current investigation reflects the substantial metabolic association between hepatic and biliary disorders within the hepatobiliary system. Gallstone disease, NAFLD, and higher all-cause mortality have also been linked in earlier epidemiological data, underscoring the problems' wider clinical importance (14). Current treatment approaches place a greater emphasis on food modification, weight control, and metabolic risk reduction as crucial elements of hepatobiliary care due to these shared metabolic characteristics (22).

These results may also be helpful in preoperative bariatric evaluation, in which nutritionists and surgeons collaborate to enhance patients' metabolic risk and body habitus prior to surgery. Even though the current study did not include bariatric surgery patients, the Western food pattern was associated with higher BMI, wider waist circumference, greater calorie intake, physical inactivity, and unfavorable hepatobiliary markers. These results highlight the importance of evaluating metabolic condition and diet quality prior to bariatric surgery. This is particularly important for patients who have both obesity and NAFLD/NASH, as a predictive model of patients with obesity and concurrent NASH, sleeve gastrectomy was linked to slower NASH progression and a reduced simulated requirement for liver transplantation when compared to medical weight management (23). As a result, food quality, obesity, and hepatobiliary condition should all be taken into account during preoperative nutritional planning.

### 4.1 Strengths

One of the strengths of this study is the thorough evaluation of diets as a result of principal component analysis that was based on a validated semi-quantitative food frequency questionnaire, which enabled the evaluation of actual world nutritional practices, as opposed to individual nutrient. The comparative case study of nonalcoholic fatty liver disease and gallstone disease is an integrated look at the concept of hepatobiliary homeostasis, which is seldom studied in the same analytical remit. The incorporation of objective clinical outcomes verified by the use of abdominal ultrasonography improves the reliability of the diagnosis, whereas the presence of detailed anthropometric, biochemical, and lifestyle information allowed to effectively adjust multivariate to the important confounders. Furthermore, stratified analyses based on central and total adiposity shed more light on the relationships found among metabolically sensitive categories. The internal validity and applicability of the results to tertiary-care populations is also strengthened by the relatively large sample size and the use of routinely gathered clinical data.

### 4.2 Limitations

There are a number of limitations that should be taken into consideration during the interpretation of the findings. The retrospective observational design does not allow causal inference and it also does not provide the possibility of establishing temporal linkages between dietary patterns and hepatobiliary outcomes. Dietary consumption was self-reported with an FFQ, which is prone to recall bias and error, but this is inherent to the majority of nutritional epidemiology studies. Ultrasonography which is considered to be clinically convenient and used extensively might under-detect mild hepatic steatosis in favor of other more sensitive imaging techniques like MRI. Furthermore, the lack of bile acid profiles, gut microbiota-related factors, and inflammatory markers in the dataset limited the ability to interpret the observed relationships mechanistically. The possibility of residual confounding is not completely eliminated since unmeasured variables such as genetic predisposition, gut microbiota composition and detailed socioeconomic markers were non-available. Additionally, socioeconomic factors, medication use, smoking status, and diabetes status were not included in the dataset and could not be incorporated into the multivariable models, which could have led to residual confounding. Another drawback is that the dataset did not contain information on prior bariatric surgery and GLP-1 receptor agonist treatment, and was not incorporated in the exclusion criteria or multivariable models. These variables are clinically pertinent as both bariatric surgery and GLP-1 receptor agonist use may improve obesity-associated metabolic status and NAFLD/NASH outcomes (24–27). As a result, it is impossible to completely rule out residual confounding from unmeasured treatment-related interventions. Consequently, it is impossible to completely rule out residual confounding from unmeasured treatment-related interventions. Furthermore, the multivariable models did not account for a number of possible confounders, including alcohol consumption, smoking status, PCOS, and steroid medication. Therefore, residual confounding cannot be ruled out, and the noticed associations should be taken carefully rather than as conclusive independent effects. Lastly, the tertiary-care outpatient clinics were used to collect the study population and this might not be generalizable to community-based or asymptomatic population. In addition, inflammatory markers, bile acid profiles, and gut microbiota-related parameters were not available in the dataset, limiting mechanistic interpretation of the observed associations.

### 4.3 Future directions

There is need to focus on prospective cohort designs in future studies to establish the temporal and possibly causal relationship between dietary habits and hepatobiliary disorders. Whole-diet trials, especially anti-inflammatory and Mediterranean-style diets, should be tested by randomized controlled trials to prove their protective effect on liver fat buildup and gallstones. Future studies that include state-of-the-art imaging tools and biomarkers of inflammation, bile acid metabolism, and oxidative stress, would enhance a better understanding of underlying biological processes. Also, genome-wide genetic risk score and gut microbiome profiling studies may assist in the identification of individuals who are most vulnerable to diet-induced hepatobiliary dysfunction, which will eventually lead to a personalized dietary approach. It will also be necessary to expand research to other groups of people and healthcare environments to enhance external validity and applicability globally.

## 5 Conclusion

In this study, dietary patterns were related to variations in metabolic indicators, lifestyle factors, and anthropometric traits. Even though Western dietary pattern was related to increased odds of NAFLD and gallstone disease in crude and partially adjusted models, these relationships were weakened and became non-significant after waist circumference, total calorie consumption, and physical inactivity were taken into account. These results imply that the observed relationships may be mostly explained by factors linked to lifestyle and obesity. As a result, the findings encourage a careful interpretation and emphasize the necessity of future research to determine if dietary habits alone increase the risk of hepatobiliary disease.

## Data Availability

The raw data supporting the conclusions of this article will be made available by the authors, without undue reservation.

## Supplementary material

Supplementary Figure S1 Scree plot.

## References

[1] Eslamparast T Tandon P Raman M. Dietary composition independent of weight loss in the management of non-alcoholic fatty liver disease. Nutrients. (2017) 9:800. doi: 10.3390/nu9080800 28933748 PMC5579594

[2] Montemayor S García S Monserrat-Mesquida M Tur JA Bouzas C. Dietary patterns, foods, and nutrients to ameliorate non-alcoholic fatty liver disease: a scoping review. Nutrients. (2023) 15:3987. doi: 10.3390/nu15183987 37764771 PMC10534915

[3] Noureddin M Zelber-Sagi S Wilkens LR. Diet associations with nonalcoholic fatty liver disease in an ethnically diverse population: the multiethnic cohort. Hepatology. (2020) 71:1940–52. doi: 10.1002/hep.30967 31553803 PMC7093243

[4] Bakhshimoghaddam F Baez D Dolatkhah N Sheikh M Poustchi H Hekmatdoost A . Which dietary patterns fend off nonalcoholic fatty liver disease? A systematic review of observational and interventional studies. BMC nutrition. (2024) 10:153. doi: 10.1186/s40795-024-00961-8 39609906 PMC11606097

[5] Zhang S Gu Y Bian S Górska MJ Zhang Q Liu L . Dietary patterns and risk of non-alcoholic fatty liver disease in adults: A prospective cohort study. Clin Nutr. (2021) 40:5373–82. doi: 10.1016/j.clnu.2021.08.021 34560608

[6] Naseri K Saadati S Asadzadeh-Aghdaei H Hekmatdoost A Sadeghi A Sobhani SR . Healthy dietary pattern reduces risk of gallstones: results of a case-control study in Iran. Int J Prev Med. (2022) 13:66. doi: 10.4103/ijpvm.IJPVM_455_19 35706852 PMC9188884

[7] Jessri M Rashidkhani B. Dietary patterns and risk of gallbladder disease: a hospital-based case-control study in adult women. J Health Popul Nutr. (2015) 33:39–49. 25995720 PMC4438647

[8] Li H Du T Miao C Deng Y Tian H Fang W. Association between dietary fiber intake and gallstones among American adults: a cross-sectional study. J Health Popul Nutr. (2025) 44:322. doi: 10.1186/s41043-025-01062-3 40898378 PMC12406361

[9] Zhang Y Zhang J Yu D Tu F Liu J Han B . Association between metabolic dysfunction associated steatotic liver disease and gallstones in the US population using propensity score matching. Sci Rep. (2025) 15:910. doi: 10.1038/s41598-025-85218-5 39762481 PMC11704231

[10] Sheikh MY Younus MF Shergill A Hasan MN. Diet and lifestyle interventions in metabolic dysfunction-associated fatty liver disease: a comprehensive review. Int J Mol Sci. (2025) 26:9625. doi: 10.3390/ijms26199625 41096891 PMC12524441

[11] Ghorbani M Hekmatdoost A Darabi Z Sadeghi A Yari Z. Dietary inflammatory index and risk of gallstone disease in Iranian women: a case-control study. BMC Gastroenterol. (2023) 23:311. doi: 10.1186/s12876-023-02943-9 37710148 PMC10500896

[12] Willett WC Sampson L Stampfer MJ Rosner B Bain C Witschi J . Reproducibility and validity of a semiquantitative food frequency questionnaire. Am J Epidemiol. (1985) 122:51–65. doi: 10.1093/oxfordjournals.aje.a114086 4014201

[13] Zelber-Sagi S Godos J Salomone F. Lifestyle changes for the treatment of nonalcoholic fatty liver disease: a review of observational studies and intervention trials. Therap Adv Gastroenterol. (2016) 9:392–407. doi: 10.1177/1756283X16638830 27134667 PMC4830109

[14] Konyn P Alshuwaykh O Dennis BB Cholankeril G Ahmed A Kim D. Gallstone disease and its association with nonalcoholic fatty liver disease, all-cause and cause-specific mortality. Clin Gastroenterol Hepatol. (2023) 21:940–8. doi: 10.1016/j.cgh.2022.04.043 35643414

[15] Martín Barraza JI Bars-Cortina D. Dietary pattern's role in hepatic epigenetic and dietary recommendations for the prevention of NAFLD. Nutrients. (2024) 16:2956. doi: 10.3390/nu16172956 39275272 PMC11396970

[16] Kotrotsios A Tasis N Angelis S Apostolopoulos AP Vlasis K Papadopoulos V . Dietary intake and cholelithiasis: a review. J Long Term Eff Med Implants. (2019) 29:317–26. doi: 10.1615/JLongTermEffMedImplants.2020034732 32749137

[17] Jin K Mi N He W Zhong R Jin B Liu Z . Dietary patterns, genetic predisposition, and risk of cholelithiasis: a large-scale prospective cohort study. Frontiers in Nutrition. (2024) 11:1469789. doi: 10.3389/fnut.2024.1469789 39399528 PMC11466834

[18] Wang Y Lu J Wen N Nie G Peng D Xiong X . The role of diet and nutrition related indicators in biliary diseases: an umbrella review of systematic review and meta-analysis. Nutr Metab. (2022) 19:51. doi: 10.1186/s12986-022-00677-1 35907868 PMC9338528

[19] Gregor A Panteva V Bruckberger S Auñon-Lopez Auñon-Lopez A Blahova S Blahova V . Energy and macronutrient restriction regulate bile acid homeostasis. J Nutr Biochem. (2024) 124:109517. doi: 10.1016/j.jnutbio.2023.109517 37925090

[20] Seaw KM Henry CJ Bi X. Relationship between non-alcoholic fatty liver disease and visceral fat measured by imaging-based body composition analysis: a systematic review. Livers. (2023) 3:463–93. doi: 10.3390/livers3030033

[21] Pang Q Zhang JY Song SD Qu K Xu XS Liu SS . Central obesity and nonalcoholic fatty liver disease risk after adjusting for body mass index. World J Gastroenterol. (2015) 21:1650–62. doi: 10.3748/wjg.v21.i5.1650 25663786 PMC4316109

[22] Romero-Gómez M Zelber-Sagi S Trenell M. Treatment of NAFLD with diet, physical activity and exercise. J Hepatol. (2017) 67:829–46. doi: 10.1016/j.jhep.2017.05.016 28545937

[23] Rouhi AD Castle RE Hoeltzel GD Williams NN Dumon KR Baimas-George M . Sleeve gastrectomy reduces the need for liver transplantation in patients with obesity and non-alcoholic steatohepatitis: a predictive model. Obes Surg. (2024) 34:1224–31. doi: 10.1007/s11695-024-07102-x 38379059

[24] Newsome PN Buchholtz K Cusi K Linder M Okanoue T Ratziu V . A placebo-controlled trial of subcutaneous semaglutide in nonalcoholic steatohepatitis. N Engl J Med. (2021) 384:1113–24. doi: 10.1056/NEJMoa2028395 33185364

[25] Armstrong MJ Gaunt P Aithal GP Barton D Hull D Parker R . Liraglutide safety and efficacy in patients with non-alcoholic steatohepatitis (LEAN): a multicentre, double-blind, randomised, placebo-controlled phase 2 study. Lancet. (2016) 387:679–90. doi: 10.1016/S0140-6736(15)00803-X 26608256

[26] Aminian A Al-Kurd A Wilson R Bena J Fayazzadeh H Singh T . Association of bariatric surgery with major adverse liver and cardiovascular outcomes in patients with biopsy-proven nonalcoholic steatohepatitis. JAMA. (2021) 326:2031–42. doi: 10.1001/jama.2021.19569 34762106 PMC8587225

[27] Fakhry TK Mhaskar R Schwitalla T Muradova E Gonzalvo JP Murr MM. Bariatric surgery improves nonalcoholic fatty liver disease: a contemporary systematic review and meta-analysis. Surg Obes Relat Dis. (2019) 15:502–11. doi: 10.1016/j.soard.2018.12.002 30683512

