# Supplementary material for: Dietary patterns and their relationship with hepatobiliary health in patients with nonalcoholic fatty liver disease and gallstone disease
Source: Front Nutr. 2026 Aug 11;13:1789238. doi: 10.3389/fnut.2026.1789238 (PMC13503224; doi:10.3389/fnut.2026.1789238)
Supplement: Supplementary file 1 [file Image_1.pdf]

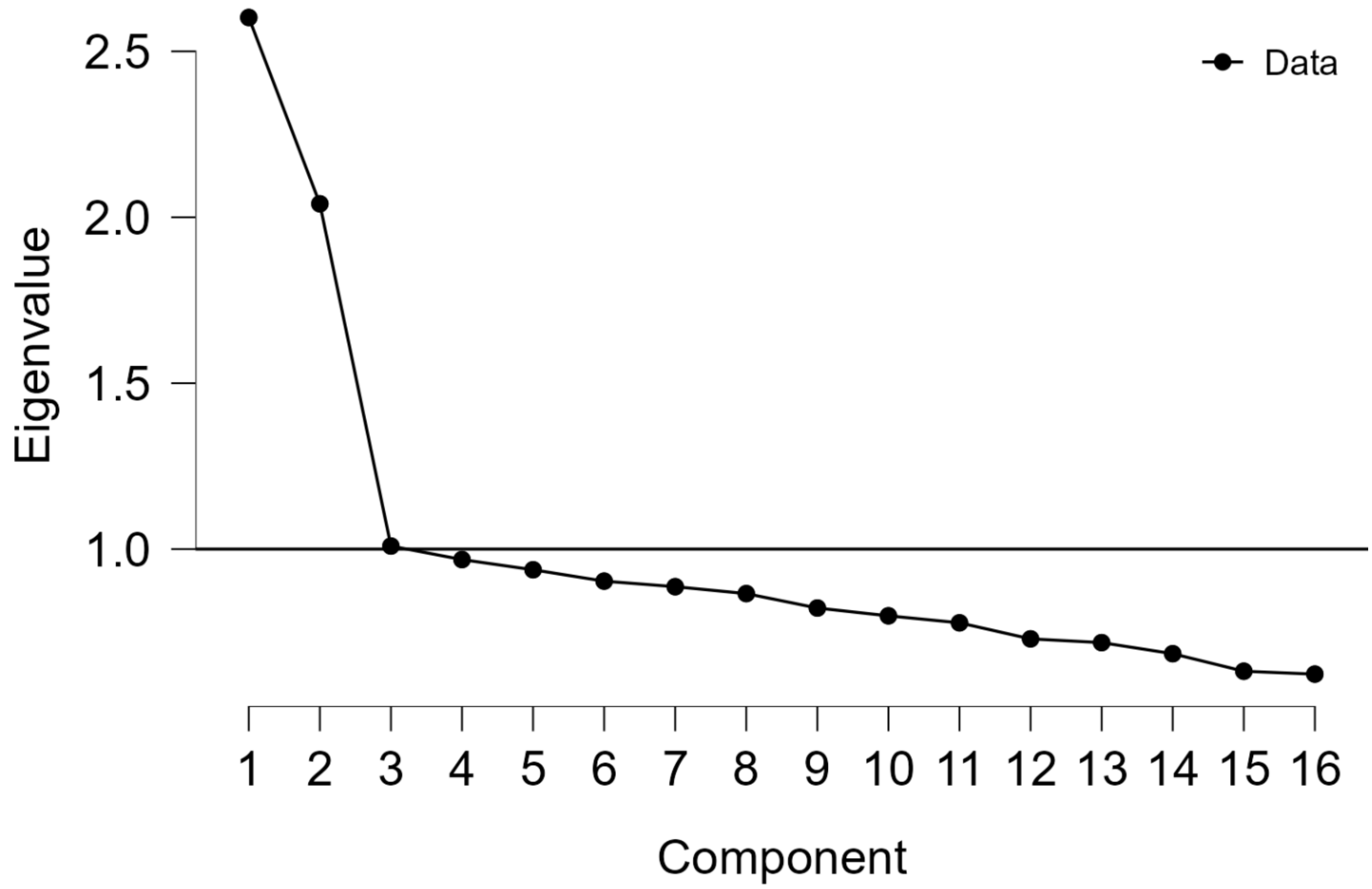

Supplementary Figure S1: Scree plot
