# Supplementary material for: Dietary patterns and their relationship with hepatobiliary health in patients with nonalcoholic fatty liver disease and gallstone disease
Source: Front Nutr. 2026 Aug 11;13:1789238. doi: 10.3389/fnut.2026.1789238 (PMC13503224; doi:10.3389/fnut.2026.1789238)
Supplement: Supplementary file 2 [file Table_1.docx]

Supplementary Table S1

**Factor loadings**

| **Food group** | **Healthy pattern (PC1)** | **Western pattern (PC2)** |
| --- | --- | --- |
| Seafood | 0.586 | — |
| Vegetables | 0.568 | — |
| Nuts/seeds | 0.534 | — |
| Fruits | 0.529 | — |
| Unsaturated oils | 0.524 | — |
| Legumes | 0.524 | — |
| Low-fat dairy | 0.512 | — |
| Whole grains | 0.502 | — |
| Refined cereals | — | 0.609 |
| Sugar-sweetened beverages | — | 0.579 |
| Salty snacks | — | 0.578 |
| Fried foods | — | 0.551 |
| High-fat dairy | — | 0.529 |
| Red/processed meat | — | 0.493 |
| Fast food | — | 0.464 |
| Sweets/desserts | — | 0.441 |
| Eigenvalue | 2.602 | 2.041 |
| Variance explained (%) | 16.3% | 12.8% |
